# A Simple Method for Discovering Druggable, Specific Glycosaminoglycan-Protein Systems. Elucidation of Key Principles from Heparin/Heparan Sulfate-Binding Proteins

**DOI:** 10.1371/journal.pone.0141127

**Published:** 2015-10-21

**Authors:** Aurijit Sarkar, Umesh R. Desai

**Affiliations:** Institute for Structural Biology, Drug Discovery & Development and Department of Medicinal Chemistry, Virginia Commonwealth University, Richmond, Virginia, United States of America; University of Patras, GREECE

## Abstract

Glycosaminoglycans (GAGs) affect human physiology and pathology by modulating more than 500 proteins. GAG-protein interactions are generally assumed to be ionic and nonspecific, but specific interactions do exist. Here, we present a simple method to identify the GAG-binding site (GBS) on proteins that in turn helps predict high specific GAG–protein systems. Contrary to contemporary thinking, we found that the electrostatic potential at basic arginine and lysine residues neither identifies the GBS consistently, nor its specificity. GBSs are better identified by considering the potential at neutral hydrogen bond donors such as asparagine or glutamine sidechains. Our studies also reveal that an unusual constellation of ionic and non-ionic residues in the binding site leads to specificity. Nature engineers the local environment of Asn45 of antithrombin, Gln255 of 3-*O*-sulfotransferase 3, Gln163 and Asn167 of 3-*O*-sulfotransferase 1 and Asn27 of basic fibroblast growth factor in the respective GBSs to induce specificity. Such residues are distinct from other uncharged residues on the same protein structure in possessing a significantly higher electrostatic potential, resultant from the local topology. In contrast, uncharged residues on nonspecific GBSs such as thrombin and serum albumin possess a diffuse spread of electrostatic potential. Our findings also contradict the paradigm that GAG-binding sites are simply a collection of contiguous Arg/Lys residues. Our work demonstrates the basis for discovering specifically interacting and druggable GAG-protein systems based on the structure of protein alone, without requiring access to any structure-function relationship data.

## Introduction

Sulfated glycosaminoglycans (GAGs), such as heparan sulfate (HS), are nature’s most enigmatic biopolymers. Although made from a linear combination of simple saccharide rings, they display a staggering range of primary sequence diversity that surpasses the range possible for equivalent chains of other biopolymers. The occurrence of HS in planaria [1], the 2^nd^ most primitive species of the animal kingdom, in a variably sulfated form closely matching that in humans suggests these biopolymers play critical roles in multiple fundamental biological processes. It is now recognized that GAGs bind to hundreds of human proteins with implications in physiological as well as pathological processes such as hemostasis and thrombosis, wound repair and inflammation, neuronal growth and amyloidogenesis, angiogenesis and cancer, defense against microbes and infection [2,3]. Yet, precious little is understood about how these unique, linear polysaccharides recognize and modulate their targets.

Considerable effort has been expended in understanding GAG–protein interactions at an atomic level. The earliest attempt to rationally deduce GAG binding sites (GBSs) on proteins was that of Cardin and Weintraub, who identified ‘XBBXBX’ and ‘XBBBXXBX’ as GAG-recognition domains, where B and X represent basic and hydropathic residues [4]. Later, these linear, α-helical or β-strand-like segments were extended to include other secondary structural elements [5,6]. However, such linear elements imply divergent evolution of GBSs, whereas the large structural diversity of GAG-binding proteins (GBPs) suggests exactly the opposite. In fact, most GBPs do not follow the simplistic Cardin and Weintraub rules. Sophisticated computational tools are being developed GBS identification [7–19]. While these approaches are successful within their set limits, a key question that remains unaddressed to date is the specificity of GAG-protein interactions [3,20,21], even though efforts have been made recently to do so [22,23]. For example, why are certain GAG–protein systems, e.g., heparin–antithrombin, highly specific [24,25], while others, e.g., heparin–thrombin [24,26,27], essentially nonspecific? More importantly, can ‘specific’ GAG–protein systems be more reliably predicted to help advance chemical biology and drug discovery?

Predicting the specificity of GAG–protein interactions is extremely challenging because of their reliance on long-range and non-directional Coulombic forces of attraction. Since multiple Arg/Lys and sulfates are involved in these interactions, many of which are redundant, most GAG–protein systems are traditionally forsaken as nonspecific. Yet, growing evidence suggests that GAGs display considerable non-ionic binding energy (10–40%) in recognizing their targets [24,28,29], which may arise from short-range and directional forces, such as hydrogen-bond(s), that induce ‘specific’ recognition. However, the exact origin of specificity among the multitude of interaction *loci* has been difficult to pinpoint.

The scientific community has historically focused on site directed mutagenesis of residues such as arginine, lysine and histidine, often present within the GBS (**Table 1**) and which will likely possess a positive charge under physiological conditions. However, GBSs also possess polar residues such as asparagine and glutamine [29]. Histidine is a special case because it may or may not be charged under physiological conditions (pK_a_ ~ 6.9) and its protonation state during GAG-binding isn’t always clear, but Asn and Gln are certainly never positively charged. Therefore, the reason for presence of Asn and Gln in GAG-binding sites remains unclear, even though it is firmly established that they interact with GAGs in many systems (**Table 1**). We asked a fundamental question: can these uncharged, polar residues have something to do with specificity?

**Table 1:** Polar residues present in various GBSs. These residues form direct interactions with GAGs, as evidenced by analysis of crystal structures.

Protein [PDB code]	Arg/Lys in GBS	Other polar residues in GBS
Antithrombin [*1tb6*, [30]]	Arg 46, 47, 129, 132, 136; Lys 114, 125, 133, 275	Asn 45
Thrombin [*1tb6*, [30]]	Arg 93, 101, 126, 165, 233; Lys 236, 240	Asn 184; Gln 256; His 87
Basic fibroblast growth factor (FGF2) [*1fq9*]	Arg 120Lys 26, 119, 125, 129, 135	Asn 27
3-O-sulfotransferase 3A1 (HS3ST3A1) [*1t8u*]	Arg 166, 190, 260, 370Lys 161, 162, 215, 259	Gln 255
3-O-sulfotransferase 1 (HS3ST1) [*3uan*]	Arg 67, 72, 197, 268, 276Lys 68, 123, 171, 173, 274	Asn 89, 167; Gln 163; His 92
2-O-sulfotransferase 1 (HS2ST1) [*4ndz*]	Arg 80, 184, 189, 190, 288Lys 111, 284, 289	Asn91, 108, 112; His 106, 140, 142

We have uncovered that uncharged residues, such as Asn or Gln, help identify the GBS and also segregate specific GAG-protein systems from nonspecific ones. Our computational results in conjunction with structural and biochemical results show that an unusual constellation of ionic and non-ionic residues constituting the GAG-binding site is necessary for high specificity interaction. These principles can help parse proteins, including those that follow the Cardin–Weintraub rule [4] as well as those that do not, based on their specificity of interaction with GAGs. Based on our findings, we propose that Asn/Gln reduce desolvation penalties during the mostly electrostatic GAG-protein interactions, in addition to hydrogen bonding to sulfates and carboxylates on the GAG. Our results challenge the traditional paradigm that GAG-binding sites on proteins are located in contiguous segments, such as helices and/or turns and that specificity arises from Arg and Lys residues. Our work is expected to fundamentally change the landscape of discovery of ‘highly specific’ GAG-binding sites on proteins, which should greatly assist with identification of druggable GAG-protein systems for designing novel GAGs and GAG mimetics as drugs.

## Results

### Electrostatic potential alone at electropositive residues does not always identify a GAG binding site

Traditionally, a cluster of electropositive residues has been assumed to form a GBS, suggesting that the electrostatic potential (*G*_*ES*_) at Arg/Lys residues should unequivocally identify GBSs. To quantitatively assess whether this expectation is correct, we calculated *G*_*ES*_ on Arg/Lys residues of representative heparin-binding proteins including antithrombin, thrombin, FGF2, HS2ST1, HS3ST1 and HS3ST3A1. The *G*_*ES*_ are represented on 2DSE plots, which we devised as new tools for easy quantitative visualization of energy at any *locus*, e.g., nitrogen donor atom of an Arg or Lys, on the protein surface in two-dimensions (see **Methods** and **Supplementary Methods**). Briefly, the position of a *locus* (such as an atom) on a protein surface is projected from 3D space onto a 2D plane to obtain a scatterplot. The area of each data point in this scatterplot is scaled in size to reflect the *G*_*ES*_ manifested at the atom by the rest of the structure. Therefore, the larger spots represent locations most conducive to binding negatively charged entities such as GAGs. Our expectation, as explained above, was that Arg/Lys of the GBS would demonstrate the highest (most negative) *G*_*ES*_.

**Fig 1:**
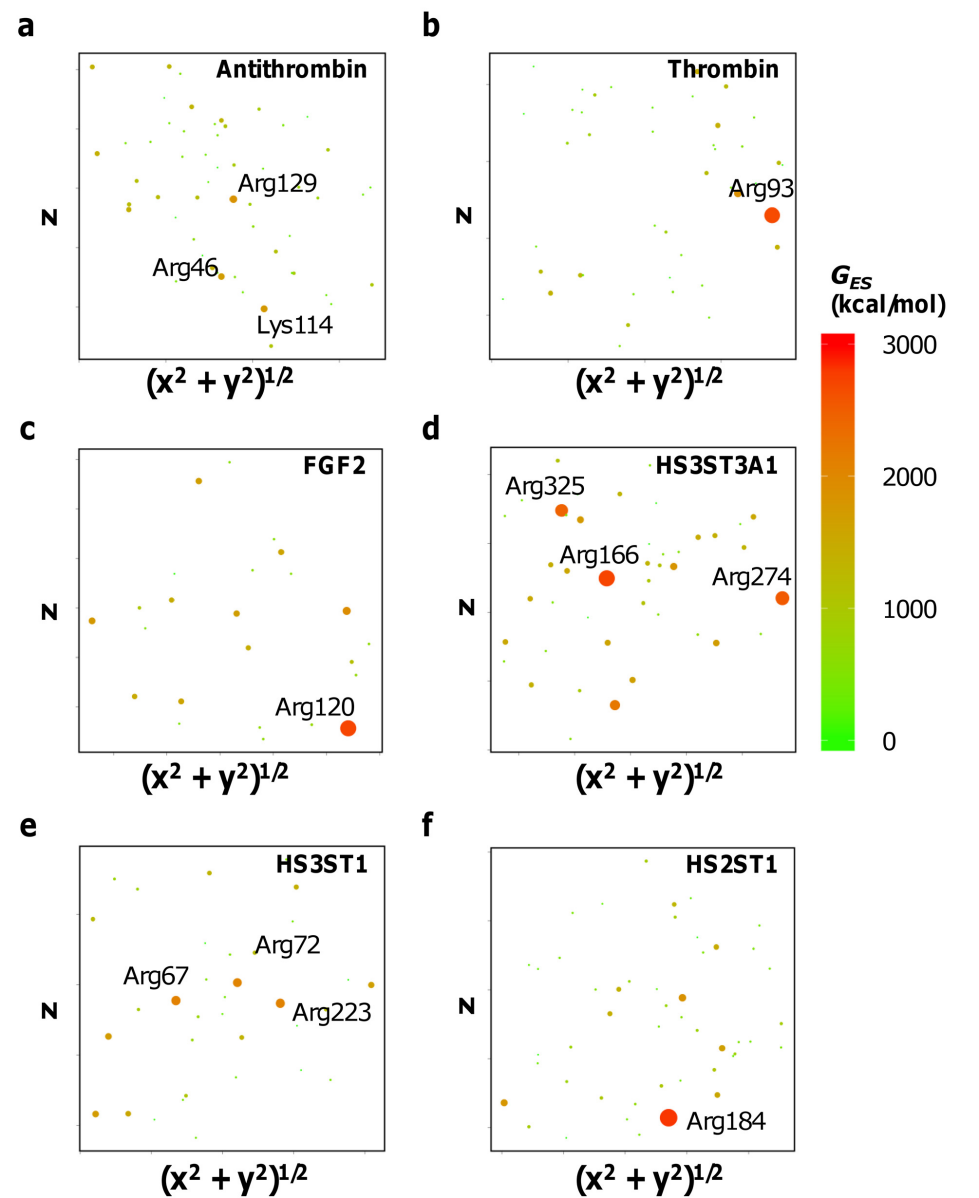
*G*_*ES*_ at arginines and/or lysines does not identify the GBS on a protein. *G*_*ES*_ for Arg/Lys residues are mapped using 2DSE plots for multiple GAG-binding proteins including **(a)** antithrombin, **(b)** thrombin, **(c)** FGF2, **(d)** HS3ST3A1, **(e)** HS3ST1 and **(f)** HS2ST1. The maps reveal that GAG-binding site Arg/Lys residues may not always possess high *G*_*ES*_ and not all Arg/Lys with high *G*_*ES*_ on a protein are part of the GAG-binding site.

Converse to our expectations, **Fig 1**reveals that not all GBS Arg/Lys (**Table 1**) carry a high *G*_*ES*_. This is in direct contrast to common empirical assumptions. For example, *G*_*ES*_ for Arg46, Lys114 and Arg129 of antithrombin were significantly higher than other basic residues present in the heparin-binding site such as Arg47, Lys125, Arg132, Lys133, Arg136 and Lys275 (**Fig 1A**). In fact, Lys114 and Arg129 are known to be crucial for heparin binding [25,31,32], but Arg46 is not. Likewise, Arg93 stands out as the only residue with high *G*_*ES*_ for thrombin, surpassing two residues (Arg97 and Arg101) known to play key role in heparin binding, (**Fig 1B**) [26,33]. Arg120 of FGF2 is only one of several, similarly contributing residues of the GBS [34,35] but demonstrates significantly higher *G*_*ES*_ (**Fig 1C**). Similar results were obtained in calculations for HS3ST3A1, HS3ST1 and HS2ST1, three enzymes of the heparan sulfate biosynthesis pathway. Although Arg166, Arg274 and Arg325 of HS3ST3A1 display high *G*_*ES*_ (**Fig 1D**), the latter two are not known to be part of the GBS [36]. Likewise, Arg223 of HS3ST1 shows strong *G*_*ES*_ (**Fig 1E**) but is not known to be part of the GBS [37]. Finally, Arg184 of HS2ST1 demonstrates high *G*_*ES*_ as well (**Fig 1F**) but is not known to be important for binding GAGs [35,38]. This implies that not all Arg/Lys residues on a GAG-binding protein’s surface carry high electrostatic potential. More importantly, not all Arg/Lys possessing high electrostatic potential contribute to GAG binding. In fact, for some proteins, multiple *loci* of high *G*_*ES*_ are observed in disparate locations, e.g., HS3ST3A1 (**Fig 1D**) and HS3ST1 (**Fig 1E**), and not all are part of the GBS [36,37]. Thus, *G*_*ES*_ at electropositive Arg and Lys residues alone does not always define GAG binding and specificity.

### Desolvation is an important factor in GAG–protein binding

**Fig 2:**
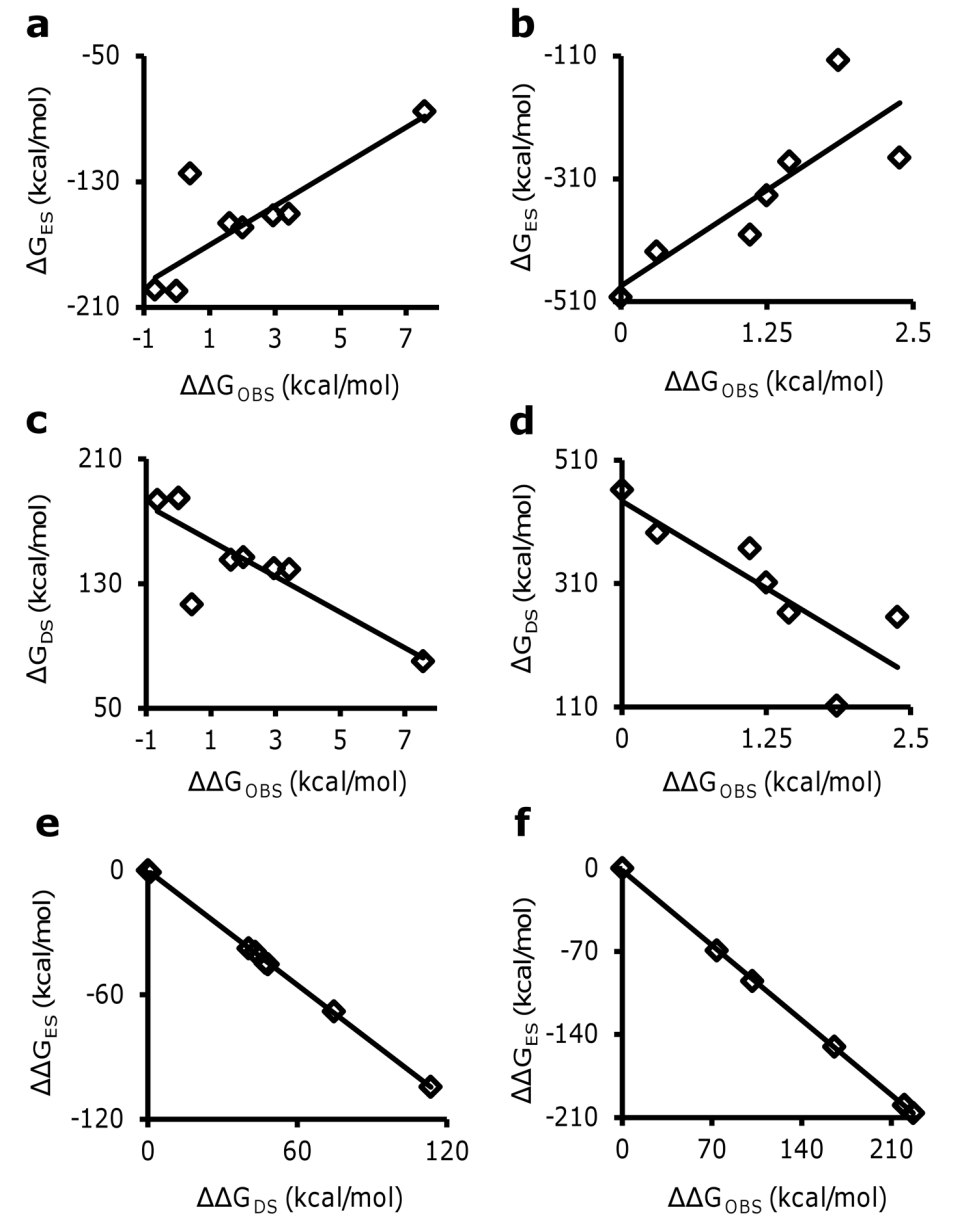
Desolvation energy is critical for quantitative analysis of GAG–protein interaction. Neither ΔG_ES_ (**a** and **b**) nor ΔG_DS_ (**c** and **d**) alone explain the change in ΔG_OBS_ for antithrombin (**a** and **c**) and thrombin (**b** and **d**) mutants studied to date. Any enthalpic gain due to electrostatics is opposed by desolvation (R^2^ = 0.99) in antithrombin **(e)** as well as in thrombin **(f)**, suggesting that desolvation is critical for quantitative analysis of GAG-protein interactions. In all cases, the correlation was found to be significant at α = 0.05.

Considering that G_ES_ alone cannot define binding specificity, we set to identify other parameters that may contribute to specificity of GAG interactions. We hypothesized that to a first approximation the overall binding energy (ΔG) of a GAG–protein system is a composite of electrostatic (ΔG_ES_) and desolvation (ΔG_DS_) free energy components. Additional terms, such as vibrational entropy and van der Waal’s energy [39,40], are likely to contribute but were not considered in this first approximation because it was our goal to identify GAG-binding sites from the structure of protein alone. Changes in vibrational entropy and van der Waal’s energy during binding will vary with nature of the cognate GAG binding partner, which precludes inclusion of these terms at this time. To test our hypothesis that ΔG_ES_ and ΔG_DS_ are the major factors determining GAG-protein interactions, we studied the antithrombin–heparin pentasaccharide and thrombin–heparin systems, for which a large body of solution experimental data is available [41–45]. For both systems, ΔG_ES_ varies linearly (R^2^~0.6–0.7) with change in free energy of GAG binding (ΔΔG_OBS_) suggesting an important role for electrostatic forces to the interaction (**Fig 2A and 2B**; see **Tables 2**and **3**). Yet, while statistically significant (*p*<0.05), the correlation is moderate at best, which quantitatively confirms that electrostatics alone does not sufficiently address binding. To assess whether the release of solvent molecules is an important contributor in GAG-protein interactions, desolvation energy (ΔG_DS_) was calculated using the Poisson-Boltzmann Surface Area (PBSA) method [46] (see **Methods**). For both proteins, ΔG_DS_ demonstrated an inverse correlation (**Fig 2C and 2D**) with ΔΔG_OBS_ (R^2^~0.6–0.7). Interestingly, electrostatic forces (ΔΔG_ES_) and desolvation forces (ΔΔG_DS_) were found to be directly opposed to each other (R^2^ = 0.99, *p*<0.05) (**Fig 2E and 2F**). More importantly, the magnitude of desolvation effects was substantial, which implies that desolvation energy cannot be discarded and is an important driver of GAG-protein association.

**Table 2:** Electrostatic interactions and desolvation energies for AT-heparin pentasaccharide complexes reported in the literature.

Mutant	ΔG_ES_ *(kcal/mol)*	ΔΔG_ES_ *(kcal/mol)*	ΔG_DS_ *(kcal/mol)*	ΔΔG_DS_ *(kcal/mol)*	K_d_ ^*b*^ *(nM)*	ΔΔG_OBS_ *(kcal/mol)*
Wt	-199.8	0	184.6	0	6	0
K125Q	-125.1	74.7	116.6	-68.0	12	0.4
K136T	-200.0	-0.25	185.0	0.4	6	0
N135A	-198.9	0.8	183.7	-0.9	2	-0.6
N135A/R129Q^*a*^	-150.7	48.2	139.2	-45.4	1800	3.4
N135A/R129H^*a*^	-151.7	47.2	139.8	-43.9	820	2.9
N135A/K114A^*a*^	-85.4	113.5	80.2	-103.5	1800000^*c*^	7.6
R132M	-156.7	43.0	145.2	-39.4	89	1.6
K133M	-159.4	40.4	147.0	-37.6	171	2.0

^*a*^ Calculated from a comparison with the N135A mutant.

^*b*^ K_d_ values were obtained from references [41–44]. **ΔΔ**G_OBS_ values were calculated using the formula **Δ**G = RTlog_e_(K_d_^1^/K_d_^2^) where K_d_^1^ is for mutant and K_d_^2^ is for wt.

^*c*^ Calculated from data available in reference [43].

**Table 3:** Effect of electrostatic interactions on binding of various thrombin mutants with heparin.

Mutant	ΔG_ES_ *(kcal/mol)*	ΔΔG_ES_ *(kcal/mol)*	ΔG_DS_ *(kcal/mol)*	ΔΔG_DS_ *(kcal/mol)*	K_d_ ^*b*^ *(nM)*	ΔΔG_OBS_ *(kcal/mol)*
Wt	-503.1	0	461.9	0	90	0
K169E	-275.9	73.8	392.4	-69.4	150	0.31
R175E	-401.6	101.5	366.8	-95.1	570	1.11
R233E	-337.3	165.8	311.5	-150.4	720	1.25
K236E	-275.9	227.2	255.5	-206.4	4800	2.38
K240E	-282.6	220.5	262.5	-199.4	1000	1.44
R233E-K240E^*a*^	-117.1	220.2	111.8	-199.7	16000	3.11

^*a*^ Calculated from comparison with the R233E mutant.

^*b*^ These values were reported in reference [45] as equilibrium constant for thrombin dissociating from the ternary antithrombin-thrombin-heparin complex. Since the mutations are at exosite II, where heparin binds (and not antithrombin), these quantify thrombin-heparin interactions. **ΔΔ**G values were calculated using the formula **Δ**G = RTlog_e_(K_d_^1^/K_d_^2^) where K_d_^1^ is for mutant and K_d_^2^ is for wt.

Since desolvation opposes electrostatic interactions, we hypothesized that residues capable of (a) producing non-ionic interactions such as hydrogen bonds (which also possess a partial covalent character to compensate for less favorable ionic interactions) with GAGs, and (b) lowering desolvation costs during such interactions, would play an important role in such interactions. Considering that GAGs possess several hydrogen bond acceptors, e.g., hydroxyls, carboxylates and sulfates, it is likely that asparagine and glutamine residues may serve as hydrogen bond donors.

### GAG-protein sites are consistently identified by *G*_*ES*_ at uncharged polar residues

It has been long known that GBSs do possess uncharged polar residues [29]. **Table 1**provides some typical examples. Their importance has mostly been construed from their presence in co-crystal structures and yet they have not been evaluated routinely by site-directed mutagenesis. However, whenever they have been, significant reduction in binding and activity are observed. For example, the Gln255Ala mutant of HS3ST3A1, a key enzyme that introduces a rare 3-sulfate group of the herpes simplex virus 1 recognition motif, demonstrates a >99% loss in activity [36]. Similarly, the Gln163Ala and Asn167Ala mutants of HS3ST1, the enzyme responsible for introduction of the 3-sulfate in unit F of DEFGH, show a ~60% drop in activity [37,47]. Likewise, Asn27, Gln123 and Gln134 of FGF2 have also been implicated in heparin binding, of which Asn27 makes a significant enthalpic contribution [34]. However, the reason for such observations has typically not been clear. Given the predominant roles played by Arg/Lys in GAG-protein binding, it seems unlikely that Asn/Gln alone would form motifs capable of binding GAGs. We predicted that a combined effect manifested by both, electropositive and uncharged residues, may provide us with clues about the roles of such residues in GAG-protein interactions.

**Fig 3:**
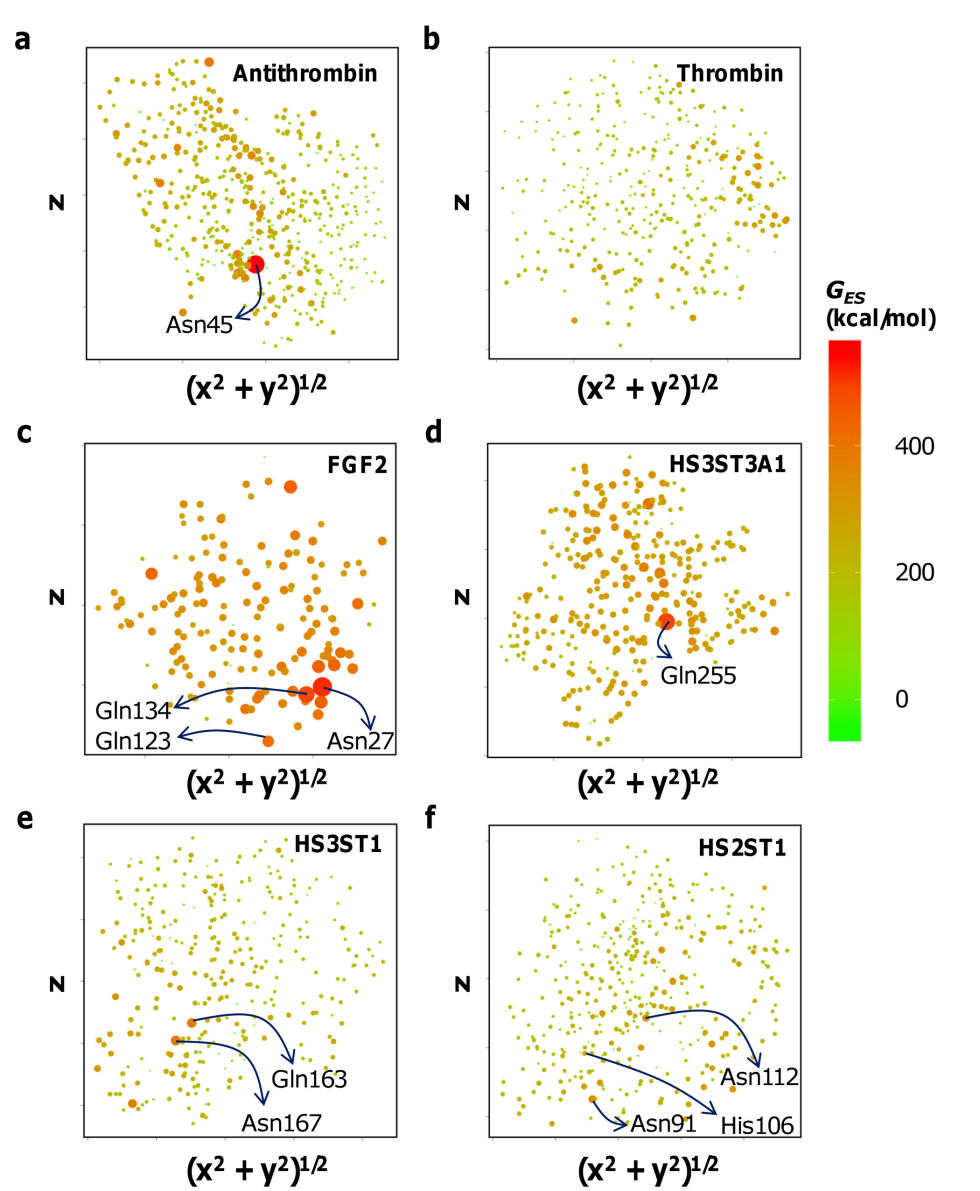
2DSE plots for *G*_*ES*_ at neutral hydrogen bond donors. GAGs bind neutral hydrogen bond donors on the protein that possess significantly high G_ES_. **(a)** Asn45 of antithrombin GAG-binding site possesses the highest G_ES_ within the structure. **(b)** In contrast, the nonspecific thrombin GAG-binding site demonstrates a diffused G_ES_. Similarly, significantly high *G*_*ES*_ are observed at **(c)** Asn27 of the FGF2 GBS; Asn27Ala mutation affects GAG-binding (ΔΔG~1.1 kcal/mol) almost as much as K125A (ΔΔG~1.7 kcal/mol), which had the largest effect, **(d)** Asn255 of the HS3ST3A1 GAG-binding site; the N255A mutant is inactive, and **(e)** Gln163 of HS3ST1; Gln163Ala mutant loses ~65% activity. **(f)** Diffused G_ES_ of HS2ST1 may represent its ability to bind low-sulfated GAGs. However, Asn91 and 112 of the HS2ST1 GAG-binding site possess a potential higher than His106, mutation of which is already known to affect GAG-binding.

Electrostatic interactions are effective at long range and therefore Arg/Lys may also affect the environment of other residues in a protein structure. For example, *G*_*ES*_ at Asn and Gln residues may be significantly altered. **Fig 3**demonstrates *G*_*ES*_ at Asn and Gln residues (and other neutral hydrogen bonding donors) in the form of 2DSE plots. While *G*_*ES*_ calculated at basic Arg and Lys residues failed to reliably identify the GBS (**Fig 1**), the same calculated for neutral hydrogen bond donors identified the GBS consistently (**Fig 3**). The highest *G*_*ES*_ for any given protein structure is always present at the GBS, as observed for antithrombin, thrombin, FGF2, HS3ST3A1, HS3ST1, HS2ST1 and serum albumin (**Fig 3**and **S1 Fig**). The identification of the GBS was independent of specific or nonspecific nature of the protein. Quite clearly, the critical positioning of basic residues near uncharged hydrogen bond donors creates a unique environment which is preferentially bound by GAGs. Thus, *G*_*ES*_ at neutral hydrogen bond donors can be called G_GAG-binding_. As will be demonstrated below, *G*_*GAG-binding*_ can segregate specific GBSs from nonspecific ones.

### Uncharged polar residues of specific GBSs possess significantly higher G_GAG-binding_ than the rest of the protein

Antithrombin, FGF2 and sulfotransferases possess specific GBSs while thrombin and serum albumin are known to be nonspecific in nature [25,27,30–32,36,37,35,38,47–49]. All these proteins possess basic and uncharged polar residues in their binding site. We can correctly identify their GBSs by calculating G_GAG-binding_ at uncharged hydrogen bond donors (*vide supra*), but can we segregate specific proteins from nonspecific ones? By first principles, a specific GBS will be an area on the protein surface that possesses significantly higher G_GAG-binding_ compared to the rest of the protein. GAGs will therefore preferentially bind to that location rather than anywhere else.

For antithrombin, the location of Asn45 possesses a much larger G_GAG-binding_ (**Fig 3A**) than any other area on its surface, indicating that GAGs will preferentially bind to it. Replacement of Asn45 with Ala, Arg or Lys completely alters the unique electrostatic environment observed in the *wt* structure (**S2 Fig**) suggesting Asn45 is a “hot spot” for binding the heparin pentasaccharide. In contrast, thrombin did not demonstrate any such unique neutral hydrogen-bond donor (**Fig 3B**) despite the presence of many basic residues in its GBS. Likewise, bovine serum albumin demonstrated no outstanding neutral donor *loci* (**S1 Fig**). It is also known to interact with heparin nonspecifically [49].

An analysis of other GAG-interactors also confirmed our ability to identify such *loci*; the 2DSE plots for neutral hydrogen-bond donors of FGF2, HS3ST3A1 and HS3ST1 show focused *G*_*GAG-binding*_ at highly localized regions (**Fig 3C, 3D and 3E**) similar to AT (**Fig 3A**). The same observations are made at various dielectric constants and by employing the CHARMM charge model [50,51], (**S3 and S4 Figs**). Note that using the more accurate CHARMM charge model gave us a better representation of HS2ST1’s specificity (**S3 Fig**). Thus, GAGs prefer to engage *loci* containing a strategically positioned neutral hydrogen bond donors near positively charged residues. If the *G*_*ES*_ at a few neutral hydrogen bond donors (*G*_*GAG-binding*_) is significantly high in comparison with other regions of the protein surface, then it is a specific GBS. 2DSE plots are a convenient way of identifying the GBS and visualizing its specificity.

**Fig 4:**
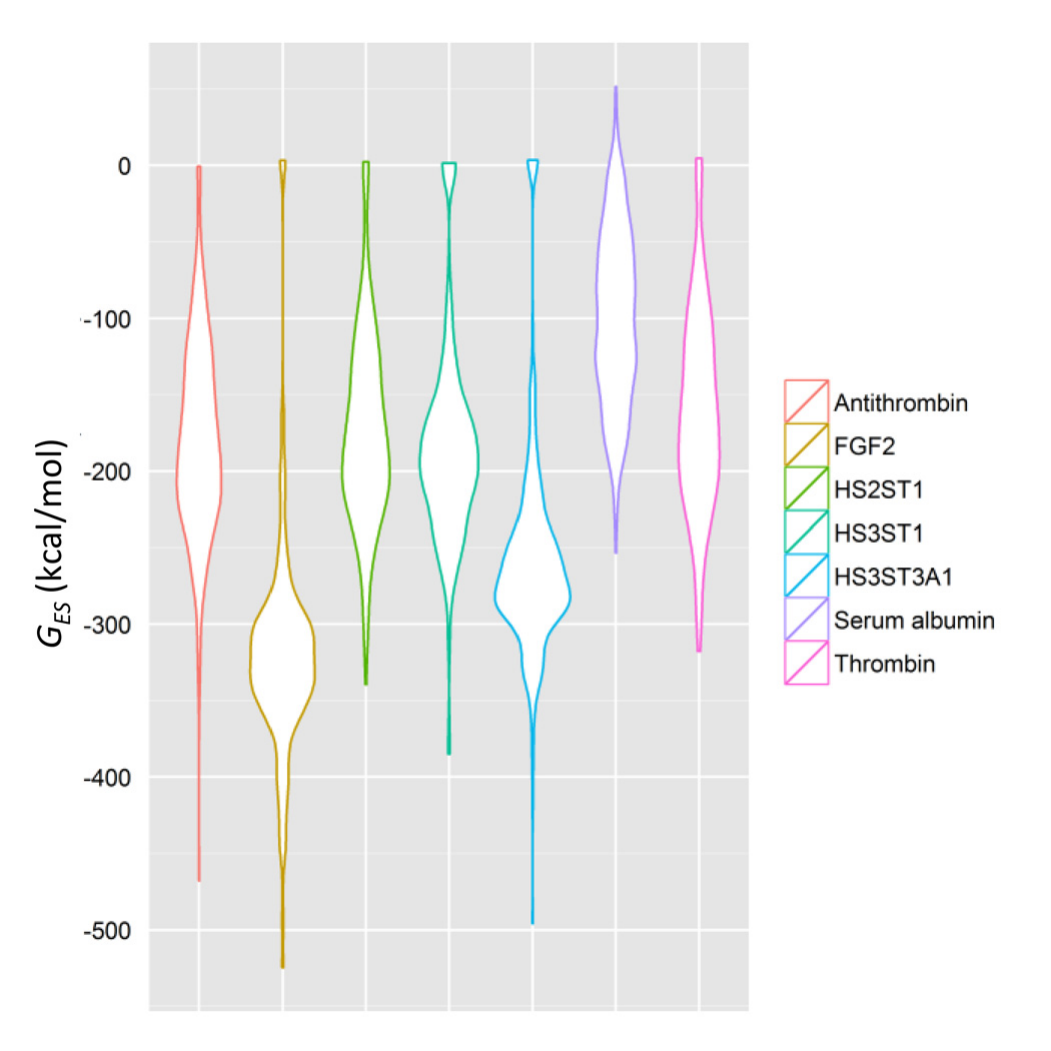
Specific proteins demonstrate unique, non-uniform distributions of electrostatic potential across neutral hydrogen-bond donors. Specific proteins such as antithrombin, FGF2, HS3ST1 and HS3ST3A1 demonstrate at least one location of electrostatic potential that deviates significantly from the mean. Nonspecific GAG-binding sites on proteins such as thrombin and serum albumin demonstrate a uniform, Gaussian distribution of the same, so no location is preferred significantly over another.

Further, a simple statistical distribution of G_GAG-binding_ across any given protein can also be used to segregate specific GBSs from nonspecific ones, as we have done in **Fig 4**. Here, G_GAG-binding_ was visualized using violin plots. The specific proteins, antithrombin, FGF2, HS3ST1 and HS3ST3A1 possess at least one location where *G*_*GAG-binding*_ is significantly higher (more negative) than the average over the entire surface, as delineated by the long tails in the plots. Conversely, the nonspecific proteins thrombin and serum albumin do not possess similar tails, which suggests *G*_*GAG-binding*_ is rather uniform across most of their surfaces and GAGs will not prefer binding at one location more than any other. This explains why they are nonspecific.

### Pharmacophoric basis for targeting specific GBSs

**Fig 5:**
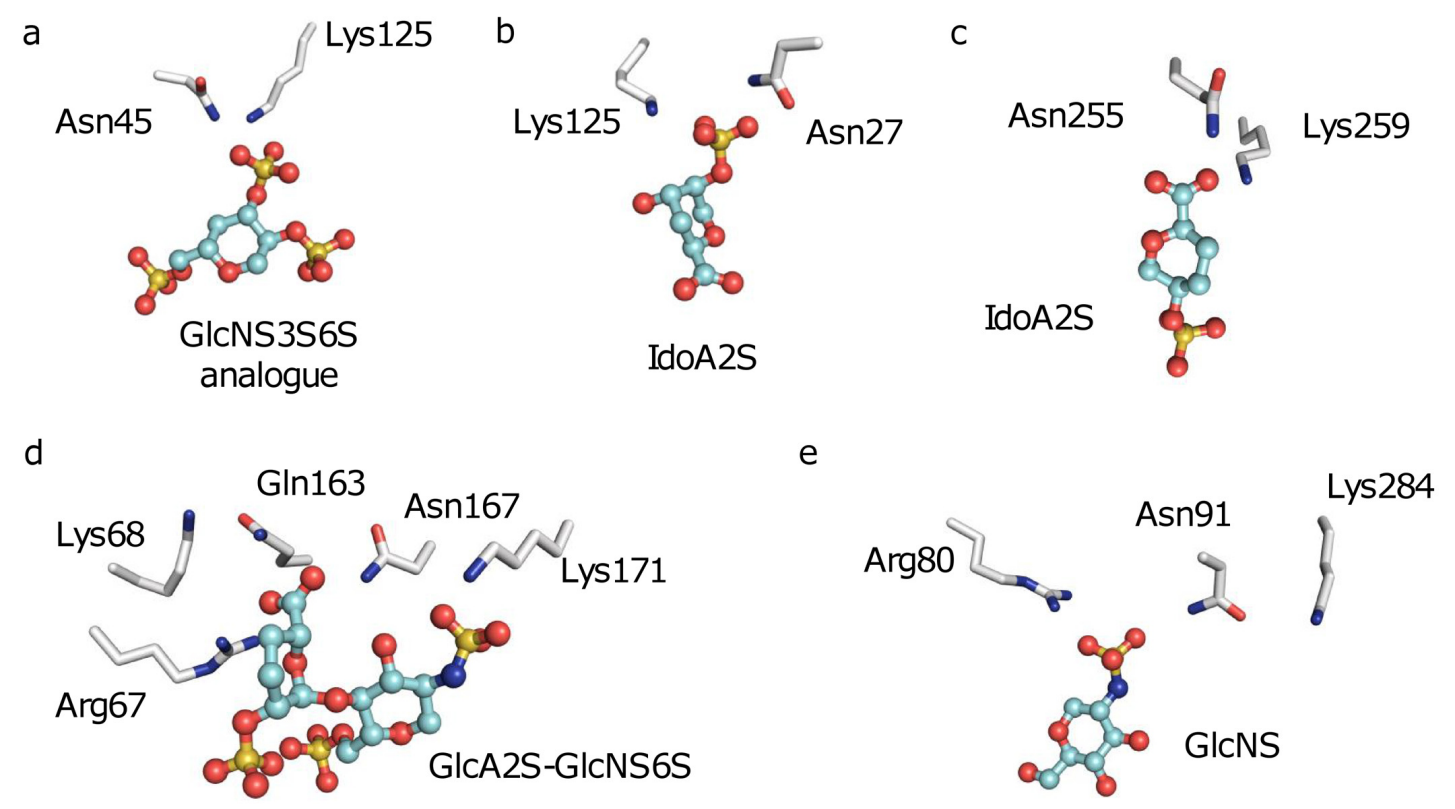
The structural basis for existence of hot spots in GBSs. Nature has designed specific GBSs by placing neutral hydrogen bond donors such as the ND2 and NE2 atoms of Asn and Gln respectively in close proximity to charged Arg or Lys residues, as seen in (a) antithrombin, (b) FGF2, (c) HS3ST3A1, (d) HS3ST1 and (e) HS2ST1. This close proximity maximizes the G_ES_ at these residues, thereby generating a specific GBS. Not all atoms are displayed, for the sake of visual clarity.

What causes a biased G_GAG-binding_ to exist in specific GAG-binding proteins? Close visualization of the antithrombin/FGF2-heparin interactions explains the origins of the unique environment surrounding Asn45 of antithrombin and Asn27 of FGF2 (**Fig 5A and 5B**). Both proteins possess positively charged basic residues very close to their respective neutral hydrogen bond donors, causing a large G_GAG-binding_ at these locations. This is the unique feature responsible for GAG-binding specificity elicited in them. Similar structural features are also observed in the sulfotransferase enzymes (**Fig 5C–5E**). Such features are a hallmark of specific GBSs.

GAG sequences that target specific proteins can be identified by considering which mono- or disaccharides will bind these pharmacophoric features. The identification of GAG sequences that may bind with specificity to a given protein is an additional challenge in GAG-protein interactions. The structural diversity of GAGs requires docking of large GAG libraries onto protein surfaces, but can be used to reasonably represent GAG-protein interactions [8,10,13,15,16,18,40,52–54]. A single residue can be crucial for interactions in GAG-protein systems, e.g. antithrombin binds only heparin containing GlcNS3S [55] and FGF2 binds only IdoA2S-containing heparin [56]. Molecular modeling studies are also most successful at identifying binding partners for such systems where evidence of specific binding exists [18,52,54]. The Combinatorial Virtual Library Screening (CVLS) approach employs an “affinity filter” and a “specificity filter” to successfully identify GAG sequences that bind proteins [15,16,52]. Similar approaches can be refined to a great extent by focused searches targeting the pharmacophoric features identified by our method. First, our method can be used to identify hot spots, followed by docking only mono- or disaccharide libraries to identify preferred motifs that bind them. The CVLS algorithm can then be used to dock focused GAG oligosaccharide libraries enriched in those preferred mono- or disaccharides. Such a comprehensive approach would pave the path for design of chemical probes and drugs.

## Discussion

Our computational results in conjunction with structural and biochemical studies indicate that (i) not all Arg/Lys carry equal and high electrostatic potential; (ii) not all Arg/Lys possessing high electrostatic potential are located in the GAG-binding site; and (iii) not all GAG-binding sites contain a neutral hydrogen-bond donor with significant electrostatic potential. On the contrary, high electrostatic potential is induced at neutral residues by nearby basic residues to generate GAG-binding sites with high specificity of recognition. In fact, we have found that considering the electrostatic potential that exists at neutral hydrogen bond donors not only depicts the correct GBS, but also reveals its specificity. Nature appears to engineer a few strategic neutral hydrogen-bond donors within the highly positively charged domain to induce specificity. At a fundamental level, this represents a major advance over the current paradigm of GAG–protein interactions, which are thought to originate only from Arg/Lys residues.

At a fundamental level, specificity of GAG–protein interaction can be of two types–‘biological’ specificity, which refers to how unique is the geometry of GAG-binding on the protein, and ‘chemical’ specificity, which refers to how unique is the sequence of a GAG that is recognized by the protein. Although most researchers assume that both biological and chemical specificities are identical for GAG-protein interaction, it is not so. For example, thrombin recognizes multiple GAG sequences, which can bind in different orientations on the protein [26] indicating that thrombin–GAG system is neither biologically specific nor chemically specific. In contrast, antithrombin recognizes a unique GAG sequence, which binds in a unique binding geometry indicating that antithrombin–GAG system is both biologically and chemically specific [24,25,28]. By the same token, proteins exhibiting intermediate forms of specificity, e.g., chemically nonspecific but biologically specific, and vice versa, may also exist. While concrete evidence of such systems is lacking, we predict that we will find such systems with increase in our ability to assess specificity of GBSs. The method we present here will certainly add to such abilities.

**Fig 6:**
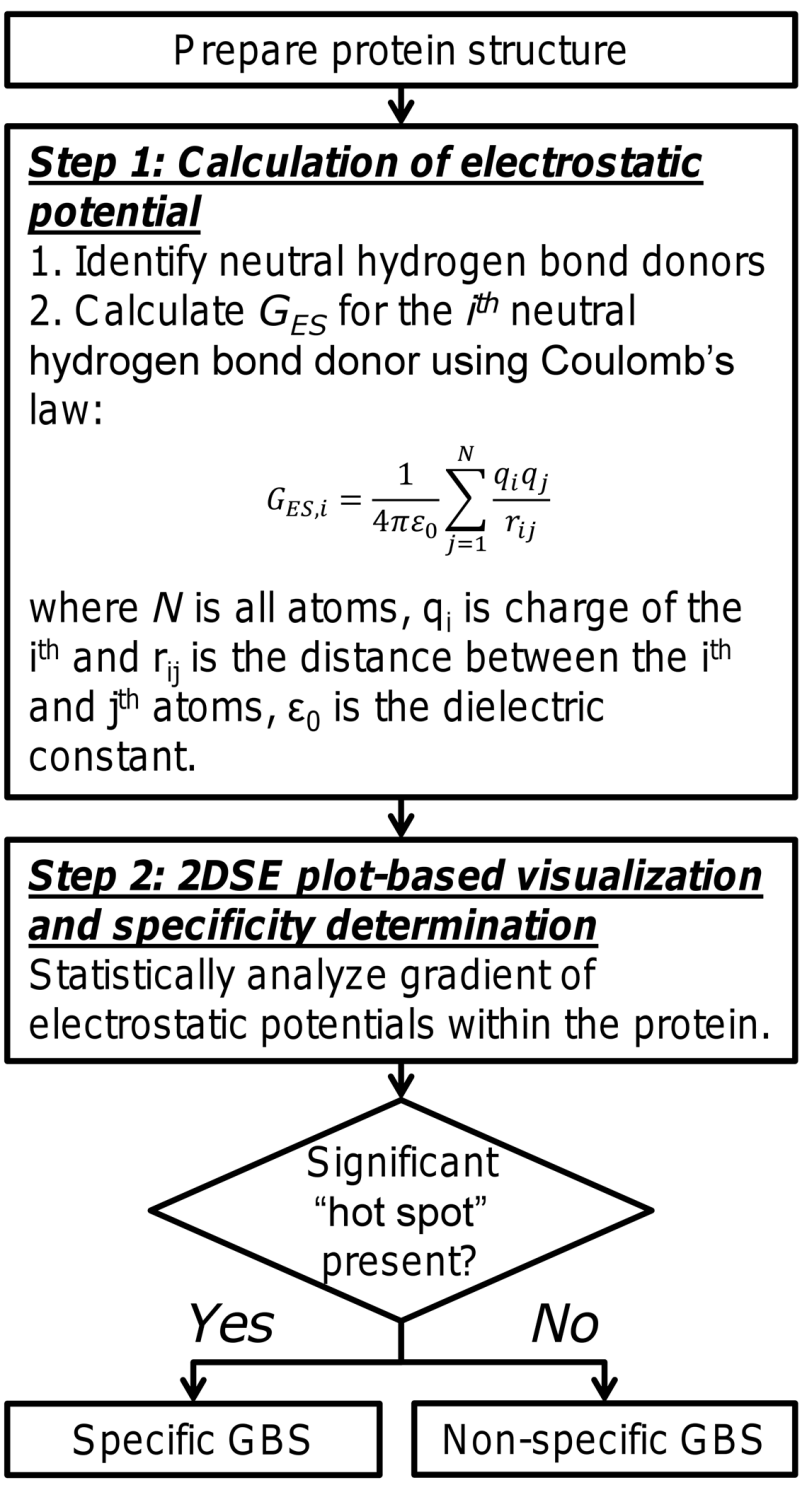
The two-step algorithm for identification of GBSs on proteins and elucidating their specificity. The process involves preparation of protein; identification of neutral hydrogen bond donors in the structure; calculation of 2DSE plots for the protein; and evaluation of ‘hot spots’ for deduction of specificity of GAG–protein interaction.

Operationally, this knowledge implies that high specificity GAG-binding sites on apo-protein structures will contain neutral hydrogen-bond donors with high (i.e., more negative) electrostatic potential compared to the rest of the protein. The advanced understanding can be used to develop a simple two-step algorithm for identification of proteins that can bind GAGs with reasonably high level of specificity (**Fig 6**).

The first step would involve calculation of electrostatic potential at every neutral hydrogen bond donor *locus* followed by statistical analysis of *G*_*ES*_ gradients to identify ‘hot spots’ i.e., neutral *loci* among multiple Arg/Lys residues that presents high electrostatic potential. Hot spots can be identified easily using 2DSE plots. Such hot spots likely engineer high GAG-binding energy through ease of desolvation penalties and hydrogen bond formation, inducing unique GAG-binding orientation and specificity of interaction. Further biophysical investigation is required to confirm the importance of desolvation energy in eliciting specificity of binding, but our ability to elucidate specific and nonspecific GBSs is already a critical advancement because it allows identification of specific GAG-binding proteins. The presence of a specific GBS on a protein likely signifies a role for GAGs in regulating that protein. Mapping specific GBSs onto biological pathways will identify novel biological roles for GAGs. Furthermore, we now have the ability to rationally target biological pathways to design GAG-based chemical probes and drugs.

Our work is expected to fundamentally change the study of GAG-protein interactions because it minimizes reliance on approximations such as linear secondary structure sequences [4] or molecular modeling/docking on regions of high positive charge density [7–19]. Because the approach can work on most high resolution structures (1.5–3.0 Å) being reported today, it is likely to ease the discovery of ‘druggable’ GAG-binding proteins, which has been a major stumbling block. In fact, our method will be of major use in rationally guiding high-throughput docking of GAG sequences [15,16,52] for identifying novel therapeutically relevant GAGs and mimetics that can specifically bind to target protein(s) and also in computational analysis of the proteome to identify the GAG interactome.

## Methods

### 2D-Surface Energy (2DSE) Plots

To the best of our knowledge, 2DSE plots have not been described in the literature. 2DSE plots enable quantitative visualization of 3D energy distribution on a protein surface in two dimensions. Here, the position of a *locus* on a protein surface is projected from 3D space onto a 2D plane to obtain a scatterplot, in which the area of the dot is scaled to reflect the *G*_*ES*_. The foundation of this novel graphical display is as follows. If ‘*i*’ is the i^th^ atom at point (*x*,*y*,*z*) in 3D space and *N* the total number of atoms in the binding site, then total energy *G* is the sum of energies at each *i* ^th^ atom located at (*x*,*y*,*z*)_*i*_ coordinate. PDB files are a typical example where the atomic (*x*,*y*,*z*) coordinates are provided for proteins. Each atom can therefore be mapped onto the abscissa–ordinate plane by plotting *(x* ^*2*^ *+y* ^*2*^ *)* ^*1/2*^ against *z*. In this projection, the expression *(x* ^*2*^ *+y* ^*2*^ *)* ^*1/2*^ is the distance of an atom/residue from the origin on the *x*-*y* plane. Further, the size of each point in the plot can be scaled relative to each other to compare their contributions towards binding. Thus, the scatter plot helps visualize the energetics (e.g., *G*_*ES*_) in 2D arising from *loci* in the 3D. Finally, each scatter point can be scaled using an exponential function to represent the change in energetics at individual *loci* in comparison to the standard, as defined by the well-established Eq 2.

$${\Delta G}_{2}-{\Delta G}_{1}\;=\;RTln\left(K_{D,2}\right)-RTln\left(K_{D,1}\right)$$ (1)

$$\frac{K_{D,2}}{K_{D,1}}=e^{\left(\frac{\Delta \Delta G}{RT}\right)}$$ (2)

Here, K_D_ represents affinity (Δ*G* = *RT* ln *K*_*D*_) and can be substituted by a factor that can be correlated with affinity. *G*_*ES*_ is one such factor known to influence GAG-protein interactions. Therefore, a direct comparison of affinities afforded by *G*_*ES*_ at two different points becomes possible using Eq 2. Typical code for generating 2DSE plots is provided in **Supplementary Methods**.

### GAG-Protein Interaction Energy and Desolvation Energy Calculation using Poisson-Boltzmann Surface Area (PBSA) Method

Electrostatic interaction energy (ΔG_ES_) and desolvation energy (ΔG_DS_) involved in antithrombin-heparin or thrombin-heparin interactions were calculated using the Poisson-Boltzmann Surface Area (PBSA) method implemented in Openeye’s ZAP toolkit [46,57]. Bondi van der Waal’s radii [58], MMFF94 [59] charges and atom-centered Gaussians [46] were used for these calculations for which we assumed an inner dielectric constant of 2 [60] and an outer dielectric constant of 80. The pdb code *1tb6* [30] provided structural information for the calculations. ΔG_ES_ was calculated using the formula ΔG_ES_ = ΔG_ES,complex_−(ΔG_ES,protein_ + ΔG_ES,GAG_). Similarly, ΔG_DS_ was calculated using ΔG_DS_ = ΔG_DS,complex_−(ΔG_DS,protein_ + ΔG_DS,GAG_).

### Statistical Analysis

Statistical analysis was performed using SigmaPlot (sigmaplot.com), or R statistical environment (r-project.org), and the ggplot2 module (ggplot2.org). Statistical significance was calculated using a two-tailed ANOVA on data found to be normal using a Shapiro-Wilk test and demonstrating constant variance. In case of unequal variances, the non-parametric Spearman rank order correlation test was used.

### Calculation of G_ES_ at Individual Residues

Multi-body *G*_*ES*_ calculations were performed using the formula in **Fig 6**implemented in the form of a Python script (**Supplementary Methods**). These calculations were performed at multiple dielectric constants, and also using the CHARMM charge model [50,51] to ensure that the results were similar.

## Supporting Information

S1 Fig 2DSE plots for serum albumin. **(a)** the *G*_*ES*_ at Arg/Lys residues reflects the relatively hydrophobic nature of albumin (it is known to bind several hydrophobic ligands to reduce their bioavailability) and **(b)** the *G*_*ES*_ at neutral hydrogen bond donors on serum albumin resembling the same map for thrombin (**Fig 1B**).
(TIF)

S2 Fig Mutation of Asn45 to Ala, Arg or Lys alters the electrostatic environment of the protein. When compared to **Fig 1A**, these 2DSE plots clearly demonstrate that the “hot spot” at Asn45 cannot exist even on mutation to Arg/Lys.
(TIF)

S3 Fig The effect of various dielectric constants on potentials. *G*_*ES*_ on neutral H-bond donors were recalculated using dielectric constants (a) 2, (b) 3, (C) 4 and (d) 10. Clearly, the trend remains exactly the same.
(TIF)

S4 Fig Effect of using CHARMM charges on potentials. CHARMM charges were used to calculate *G*_*ES*_ on neutral hydrogen bond donors for all the cases. Clearly, while the *G*_*ES*_ value may change, there is still a clear distinction between specific and non-specific proteins. However, HS2ST1 now seems far more specific than with MMFF94 charges, in line with expectations that the enzyme will possess a specific GBS.
(TIF)

S1 File An R script that creates 2DSE plots (R)

S2 File The GAG-Binding Site Predictor written in python. NOTE: This will require file 3 installed properly to function.
(PY) Click here for additional data file.

S3 File A short molecule input/output module written in python. (PY)

## References

[1] Yamada S, Sugahara K, Ozbek S. Evolution of glycosaminoglycans: Comparative biochemical study. Commun Integr Biol. 2011;4: 150–8. 10.4161/cib.4.2.14547 21655428 PMC3104567

[2] Ori A, Wilkinson MC, Fernig DG. A systems biology approach for the investigation of the heparin/heparan sulfate interactome. J Biol Chem. 2011;286: 19892–904. 10.1074/jbc.M111.228114 21454685 PMC3103365

[3] Raman R, Sasisekharan V, Sasisekharan R. Structural insights into biological roles of protein-glycosaminoglycan interactions. Chem Biol. 2005;12: 267–77. 10.1016/j.chembiol.2004.11.020 15797210

[4] Cardin A, Weintraub H. Molecular modeling of protein-glycosaminoglycan interactions. Arter Throm Vas. 1989;9: 21–32. 10.1161/01.ATV.9.1.21 2463827

[5] Sobel M, Soler DF, Kermode JC, Harris RB. Localization and characterization of a heparin binding domain peptide of human von Willebrand factor. J Biol Chem. 1992;267: 8857–62. 1577724

[6] Hileman RE, Fromm JR, Weiler JM, Linhardt RJ. Glycosaminoglycan-protein interactions: definition of consensus sites in glycosaminoglycan binding proteins. Bioessays. 1998;20: 156–167. 10.1002/(SICI)1521-1878(199802)20:2<156::AID-BIES8>3.0.CO;2-R 9631661

[7] Forster M, Mulloy B. Computational approaches to the identification of heparin-binding sites on the surfaces of proteins. Biochem Soc Trans. 2006;34: 431–4. 10.1042/BST0340431 16709179

[8] Mulloy B, Forster MJ. Application of drug discovery software to the identification of heparin-binding sites on protein surfaces: a computational survey of the 4-helix cytokines. Mol Simul. 2008;34: 481–489. 10.1080/08927020701784754

[9] Gandhi NS, Coombe DR, Mancera RL. Platelet endothelial cell adhesion molecule 1 (PECAM-1) and its interactions with glycosaminoglycans: 1. Molecular modeling studies. Biochemistry. 2008;47: 4851–62. 10.1021/bi702455e 18393439

[10] Gandhi NS, Mancera RL. Prediction of heparin binding sites in bone morphogenetic proteins (BMPs). Biochim Biophys Acta. Elsevier B.V.; 2012;1824: 1374–81. 10.1016/j.bbapap.2012.07.002 22824487

[11] Agostino M, Mancera RL, Ramsland PA, Yuriev E. AutoMap: a tool for analyzing protein-ligand recognition using multiple ligand binding modes. J Mol Graph Model. Elsevier Inc.; 2013;40: 80–90. 10.1016/j.jmgm.2013.01.001 23376613

[12] Agostino M, Gandhi NS, Mancera RL. Development and application of site mapping methods for the design of glycosaminoglycans. Glycobiology. 2014;24: 840–51. 10.1093/glycob/cwu045 24859723

[13] Mottarella SE, Beglov D, Beglova N, Nugent MA, Kozakov D, Vajda S. Docking server for the identification of heparin binding sites on proteins. J Chem Inf Model. 2014;54: 2068–78. 10.1021/ci500115j 24974889 PMC4184157

[14] Rogers CJ, Clark PM, Tully SE, Abrol R, Garcia KC, Goddard WA, et al Elucidating glycosaminoglycan-protein-protein interactions using carbohydrate microarray and computational approaches. Proc Natl Acad Sci U S A. 2011;108: 9747–52. 10.1073/pnas.1102962108 21628576 PMC3116396

[15] Raghuraman A, Mosier PD, Desai UR. Finding a needle in a haystack: development of a combinatorial virtual screening approach for identifying high specificity heparin/heparan sulfate sequence(s). J Med Chem. 2006;49: 3553–62. 10.1021/jm060092o 16759098 PMC2516555

[16] Raghuraman A, Mosier PD, Desai UR. Understanding Dermatan Sulfate-Heparin Cofactor II Interaction through Virtual Library Screening. ACS Med Chem Lett. 2010;1: 281–285. 10.1021/ml100048y 20835364 PMC2936258

[17] Torrent M, Nogués MV, Andreu D, Boix E. The “CPC clip motif”: a conserved structural signature for heparin-binding proteins. PLoS One. 2012;7: e42692 10.1371/journal.pone.0042692 22880084 PMC3412806

[18] Samsonov SA, Gehrcke J, Pisabarro MT. Flexibility and explicit solvent in molecular-dynamics-based docking of protein-glycosaminoglycan systems. J Chem Inf Model. 2014;54: 582–92. 10.1021/ci4006047 24479827

[19] Samsonov SA, Bichmann L, Pisabarro MT. Coarse-Grained Model of Glycosaminoglycans. J Chem Inf Model. 2014; 10.1021/ci500669w 25490039

[20] Spillmann D, Lindahl U. Glycosaminoglycan-protein interactions: a question of specificity. Curr Opin Struct Biol. 1994;4: 677–682. 10.1016/S0959-440X(94)90165-1 29455055

[21] Kreuger J, Spillmann D, Li J, Lindahl U. Interactions between heparan sulfate and proteins: the concept of specificity. J Cell Biol. 2006;174: 323–7. 10.1083/jcb.200604035 16880267 PMC2064228

[22] Muñoz-García JC, Chabrol E, Vivès RR, Thomas A, de Paz JL, Rojo J, et al Langerin–Heparin Interaction: Two Binding Sites for Small and Large Ligands As Revealed by a Combination of NMR Spectroscopy and Cross-Linking Mapping Experiments. J Am Chem Soc. 2015;137: 4100–4110. 10.1021/ja511529x 25747117

[23] Munoz-Garcia JC, Garcia-Jimenez MJ, Carrero P, Canales a., Jimenez-Barbero J, Martin-Lomas M, et al Importance of the polarity of the glycosaminoglycan chain on the interaction with FGF-1. Glycobiology. 2014;24: 1004–1009. 10.1093/glycob/cwu071 25015527

[24] Mosier PD, Krishnasamy C, Kellogg GE, Desai UR. On the specificity of heparin/heparan sulfate binding to proteins. Anion-binding sites on antithrombin and thrombin are fundamentally different. PLoS One. 2012;7: e48632 10.1371/journal.pone.0048632 23152789 PMC3495972

[25] Jin L, Abrahams JP, Skinner R, Petitou M, Pike RN, Carrell RW. The anticoagulant activation of antithrombin by heparin. Proc Natl Acad Sci U S A. 1997;94: 14683–8. 9405673 10.1073/pnas.94.26.14683 PMC25092

[26] Carter WJ, Cama E, Huntington J a. Crystal structure of thrombin bound to heparin. J Biol Chem. 2005;280: 2745–9. 10.1074/jbc.M411606200 15548541

[27] Olson ST, Halvorson HR, Björk I. Quantitative characterization of the thrombin-heparin interaction. Discrimination between specific and nonspecific binding models. J Biol Chem. 1991;266: 6342–6352. 2007587

[28] Desai UR, Petitou M, Bjork I, Olson ST. Mechanism of Heparin Activation of Antithrombin. Role of individual residues of the pentasaccharide activating sequence in the recognition of native and activated states of antithrombin. J Biol Chem. 1998;273: 7478–7487. 10.1074/jbc.273.13.7478 9516447

[29] Xu D, Esko JD. Demystifying heparan sulfate-protein interactions. Annu Rev Biochem. 2014;83: 129–57. 10.1146/annurev-biochem-060713-035314 24606135 PMC7851832

[30] Li W, Johnson DJD, Esmon CT, Huntington JA. Structure of the antithrombin-thrombin-heparin ternary complex reveals the antithrombotic mechanism of heparin. Nat Struct Mol Biol. 2004;11: 857–62. 10.1038/nsmb811 15311269

[31] Schedin-Weiss S, Arocas V, Bock SC, Olson ST, Björk I. Specificity of the basic side chains of Lys114, Lys125, and Arg129 of antithrombin in heparin binding. Biochemistry. 2002;41: 12369–76. 12369826 10.1021/bi020406j

[32] Desai UR. New antithrombin-based anticoagulants. Med Res Rev. 2004;24: 151–81. 10.1002/med.10058 14705167

[33] He X, Ye J, Esmon CT, Rezaie a R. Influence of Arginines 93, 97, and 101 of thrombin to its functional specificity. Biochemistry. 1997;36: 8969–76. 10.1021/bi9704717 9220985

[34] Thompson LD, Pantoliano MW, Springer BA. Energetic characterization of the basic fibroblast growth factor-heparin interaction: identification of the heparin binding domain. Biochemistry. 1994;33: 3831–40. 8142385 10.1021/bi00179a006

[35] Schlessinger J, Plotnikov AN, Ibrahimi OA, Eliseenkova A V, Yeh BK, Yayon A, et al Crystal structure of a ternary FGF-FGFR-heparin complex reveals a dual role for heparin in FGFR binding and dimerization. Mol Cell. 2000;6: 743–50. 11030354 10.1016/s1097-2765(00)00073-3

[36] Moon AF, Edavettal SC, Krahn JM, Munoz EM, Negishi M, Linhardt RJ, et al Structural analysis of the sulfotransferase (3-o-sulfotransferase isoform 3) involved in the biosynthesis of an entry receptor for herpes simplex virus 1. J Biol Chem. 2004;279: 45185–93. 10.1074/jbc.M405013200 15304505 PMC4114238

[37] Moon AF, Xu Y, Woody SM, Krahn JM, Linhardt RJ, Liu J, et al Dissecting the substrate recognition of 3-O-sulfotransferase for the biosynthesis of anticoagulant heparin. Proc Natl Acad Sci U S A. 2012;109: 5265–70. 10.1073/pnas.1117923109 22431632 PMC3325653

[38] Liu C, Sheng J, Krahn JM, Perera L, Xu Y, Hsieh P-H, et al Molecular mechanism of substrate specificity for heparan sulfate 2-O-sulfotransferase. J Biol Chem. 2014;289: 13407–18. 10.1074/jbc.M113.530535 24652287 PMC4036349

[39] Mascotti DP, Lohman TM. Thermodynamics of Charged Oligopeptide-Heparin Interactions. Biochemistry. 1995;34: 2908–2915. 10.1021/bi00009a022 7893705

[40] Gandhi NS, Mancera RL. Free energy calculations of glycosaminoglycan—Protein interactions. Glycobiology. 2009;19: 1103–1115. 10.1093/glycob/cwp101 19643843

[41] Kridel SJ, Chan WW, Knauer DJ. Requirement of Lysine Residues Outside of the Proposed Pentasaccharide Binding Region for High Affinity Heparin Binding and Activation of Human Antithrombin III. J Biol Chem. 1996;271: 20935–20941. 10.1074/jbc.271.34.20935 8702852

[42] Desai U, Swanson R, Bock SC, Bjork I, Olson ST. Role of arginine 129 in heparin binding and activation of antithrombin. J Biol Chem. 2000;275: 18976–84. 10.1074/jbc.M001340200 10764763

[43] Arocas V, Bock SC, Raja S, Olson ST, Bjork I. Lysine 114 of antithrombin is of crucial importance for the affinity and kinetics of heparin pentasaccharide binding. J Biol Chem. 2001;276: 43809–17. 10.1074/jbc.M105294200 11567021

[44] Meagher JL, Huntington JA, Fan B, Gettins PGW. Role of Arginine 132and Lysine 133in Heparin Binding to and Activation of Antithrombin. J Biol Chem. 1996;271: 29353–29358. 10.1074/jbc.271.46.29353 8910598

[45] Gan ZR, Li Y, Chen Z, Lewis SD, Shafer JA. Identification of basic amino acid residues in thrombin essential for heparin-catalyzed inactivation by antithrombin III. J Biol Chem. 1994;269: 1301–5. 8288594

[46] Grant JA, Pickup BT, Nicholls A. A smooth permittivity function for Poisson-Boltzmann solvation methods. J Comput Chem. 2001;22: 608–640. 10.1002/jcc.1032

[47] Edavettal SC, Lee KA, Negishi M, Linhardt RJ, Liu J, Pedersen LC. Crystal structure and mutational analysis of heparan sulfate 3-O-sulfotransferase isoform 1. J Biol Chem. 2004;279: 25789–97. 10.1074/jbc.M401089200 15060080

[48] Olson ST, Bjork I. Predominant Contribution of Surface Approximation to the Mechanism of Heparin Acceleration of the Antithrombin-Thrombin Reaction. Elucidation from salt concentration effects. J Biol Chem. 1991;266: 6353–6364. 2007588

[49] Hattori T, Kimura K, Seyrek E, Dubin PL. Binding of bovine serum albumin to heparin determined by turbidimetric titration and frontal analysis continuous capillary electrophoresis. Anal Biochem. 2001;295: 158–67. 10.1006/abio.2001.5129 11488617

[50] Brooks BR, Brooks CL III, Mackerell AD, Nilsson L, Petrella RJ, Roux B, et al CHARMM: The biomolecular simulation program. J Comput Chem. 2009;30: 1545–1614. 10.1002/jcc.21287 19444816 PMC2810661

[51] MacKerell AD, Bashford D, Bellott M, Dunbrack RL, Evanseck JD, Field MJ, et al All-atom empirical potential for molecular modeling and dynamics studies of proteins. J Phys Chem B. 1998;102: 3586–616. 10.1021/jp973084f 24889800

[52] Sankaranarayanan NV, Desai UR. Toward a robust computational screening strategy for identifying glycosaminoglycan sequences that display high specificity for target proteins. Glycobiology. 2014;0: 1–11. 25049239 10.1093/glycob/cwu077 PMC4296175

[53] Sapay N, Cabannes E, Petitou M, Imberty A. Molecular modeling of the interaction between heparan sulfate and cellular growth factors: bringing pieces together. Glycobiology. 2011;21: 1181–93. 10.1093/glycob/cwr052 21572110

[54] Boothello RS, Sarkar A, Tran VM, Nguyen TKN, Sankaranarayanan NV, Mehta AY, et al Chemoenzymatically Prepared Heparan Sulfate Containing Rare 2-O-Sulfonated Glucuronic Acid Residues. ACS Chem Biol. 2015;10: 1485–1494. 10.1021/acschembio.5b00071 25742429

[55] Atha DH, Lormeau JC, Petitou M, Rosenberg RD, Choay J. Contribution of monosaccharide residues in heparin binding to antithrombin III. Biochemistry. 1985;24: 6723–9. 4084555 10.1021/bi00344a063

[56] Maccarana M, Casu B, Lindahl U. Minimal sequence in heparin/heparan sulfate required for binding of basic fibroblast growth factor. J Biol Chem. 1994;269: 3903 8106436

[57] Gilson MK, Rashin A, Fine R, Honig B. On the calculation of electrostatic interactions in proteins. J Mol Biol. 1985;184: 503–516. 4046024 10.1016/0022-2836(85)90297-9

[58] Bondi A. van der Waals Volumes and Radii. J Phys Chem. 1964;68: 441–451. 10.1021/j100785a001

[59] Halgren TA. Merck molecular force field. II. MMFF94 van der Waals and electrostatic parameters for intermolecular interactions. J Comput Chem. 1996;17: 520–552. 10.1002/(SICI)1096-987X(199604)17:5/6<520::AID-JCC2>3.0.CO;2-W

[60] Ren P, Chun J, Thomas DG, Schnieders MJ, Marucho M, Zhang J, et al Biomolecular electrostatics and solvation: a computational perspective. Q Rev Biophys. 2012;45: 427–91. 10.1017/S003358351200011X 23217364 PMC3533255

