# Supplementary figures and images for: A Simple Method for Discovering Druggable, Specific Glycosaminoglycan-Protein Systems. Elucidation of Key Principles from Heparin/Heparan Sulfate-Binding Proteins
Source: PLoS One. 2015 Oct 21;10(10):e0141127. doi: 10.1371/journal.pone.0141127 (PMC4619353; doi:10.1371/journal.pone.0141127)

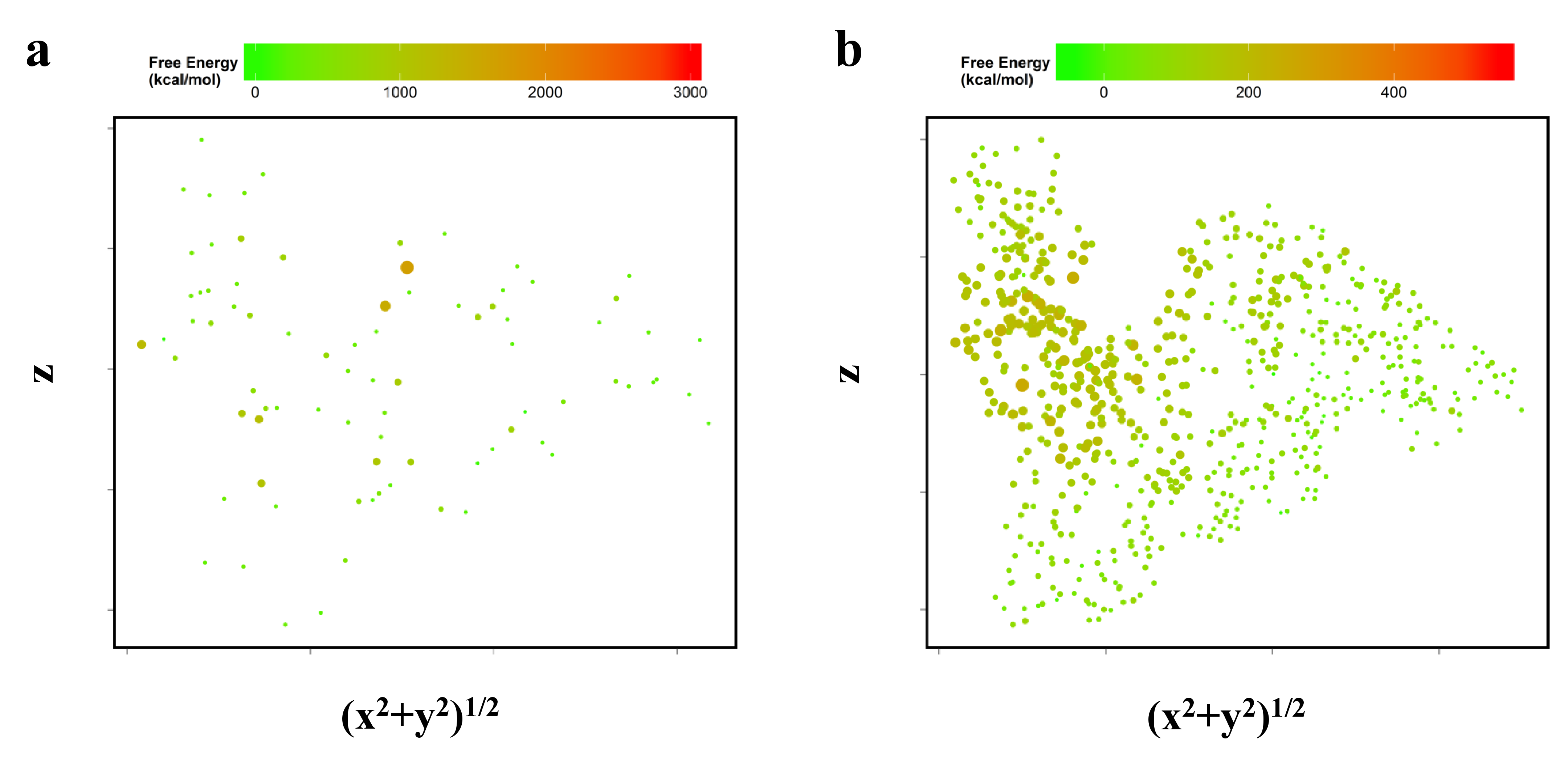

Supplement: S1 Fig — (a) the G ES at Arg/Lys residues reflects the relatively hydrophobic nature of albumin (it is known to bind several hydrophobic ligands to reduce their bioavailability) and (b) the G ES at neutral hydrogen bond donors on serum albumin resembling the same map for thrombin (Fig 1B). (TIF) [file pone.0141127.s001.tif]

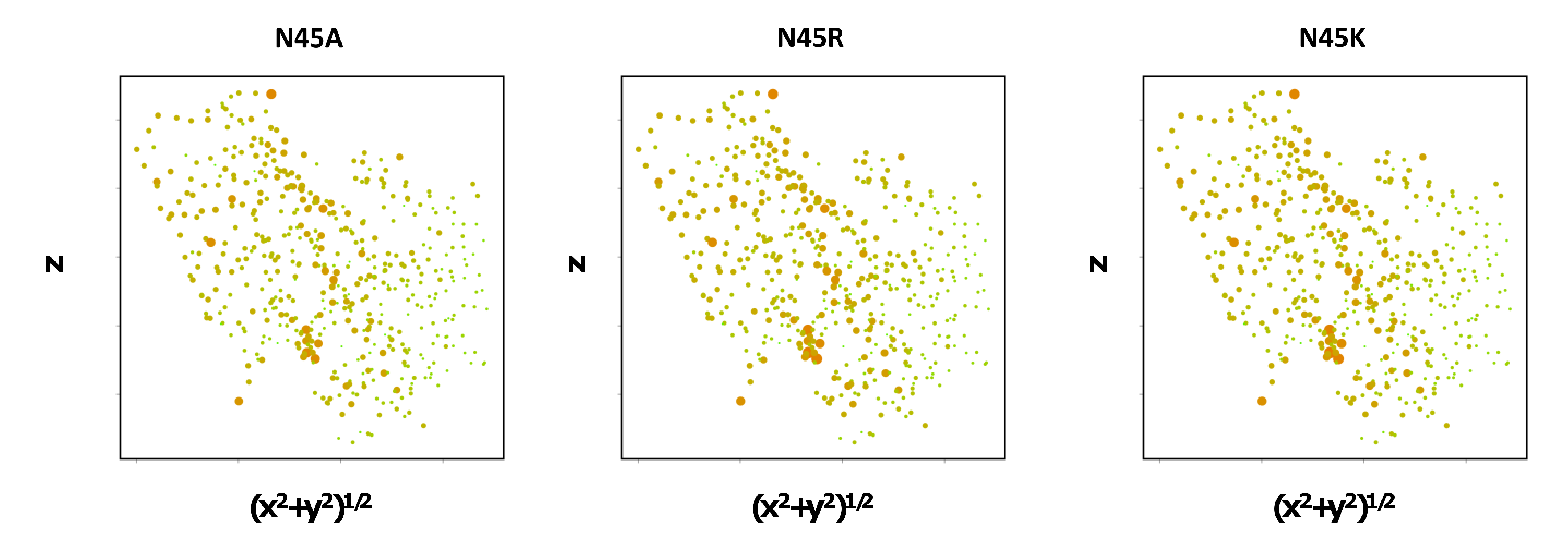

Supplement: S2 Fig — When compared to Fig 1A, these 2DSE plots clearly demonstrate that the “hot spot” at Asn45 cannot exist even on mutation to Arg/Lys. (TIF) [file pone.0141127.s002.tif]

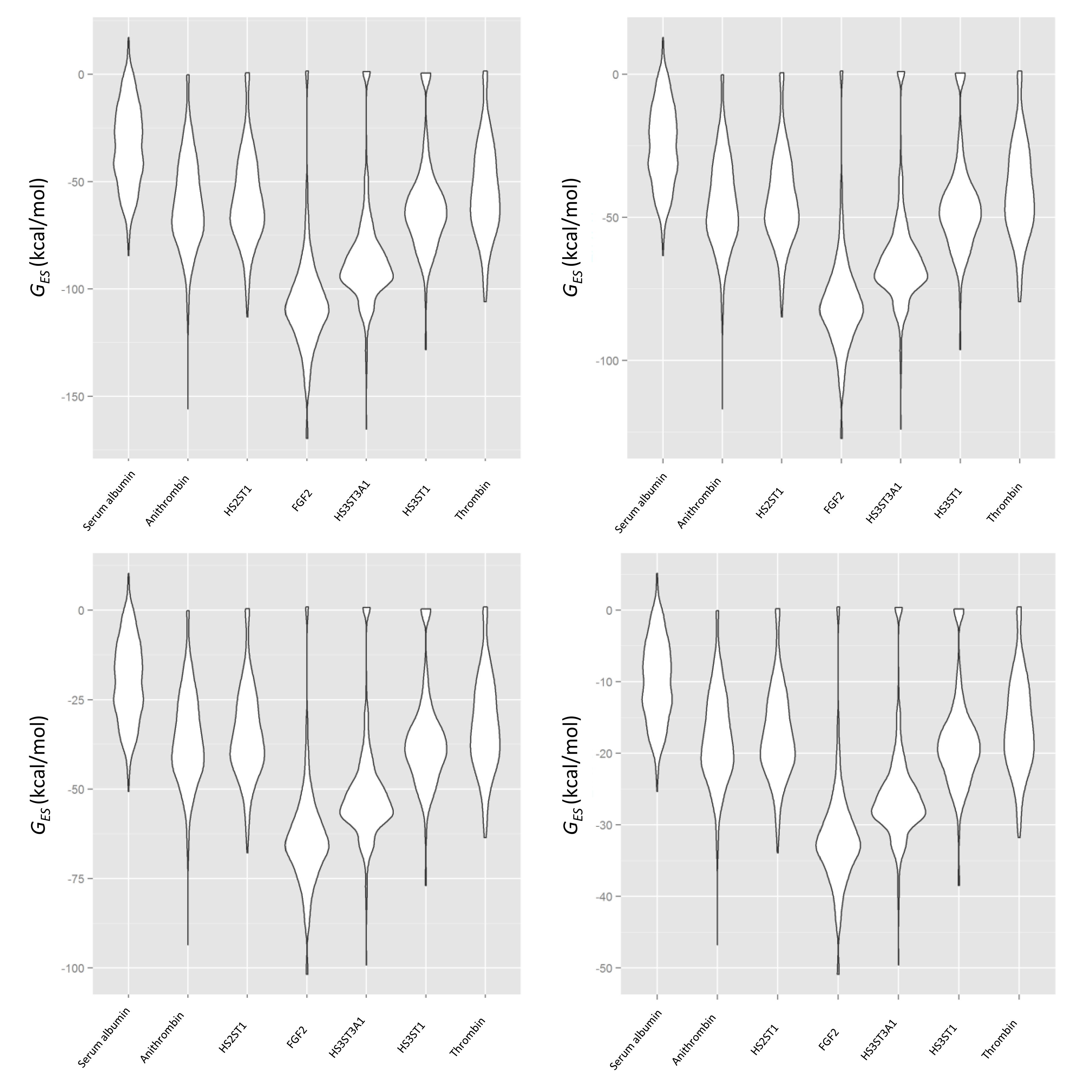

Supplement: S3 Fig — G ES on neutral H-bond donors were recalculated using dielectric constants (a) 2, (b) 3, (C) 4 and (d) 10. Clearly, the trend remains exactly the same. (TIF) [file pone.0141127.s003.tif]

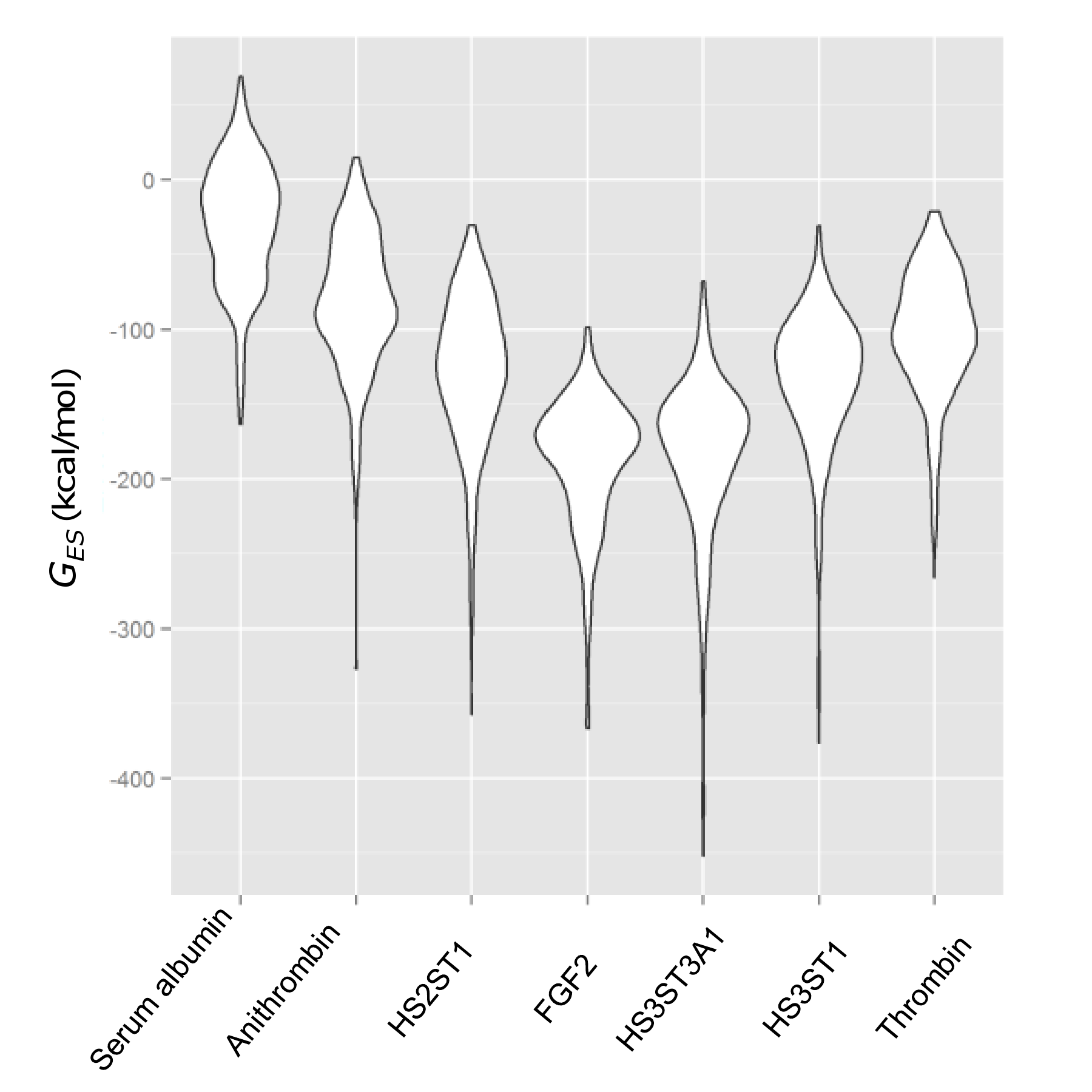

Supplement: S4 Fig — CHARMM charges were used to calculate G ES on neutral hydrogen bond donors for all the cases. Clearly, while the G ES value may change, there is still a clear distinction between specific and non-specific proteins. However, HS2ST1 now seems far more specific than with MMFF94 charges, in line with expectations that the enzyme will possess a specific GBS. (TIF) [file pone.0141127.s004.tif]
